# Prevalence of Cardiovascular Risk Factors in Smokers Versus Non-smokers: A Cross-Sectional Analysis

**DOI:** 10.7759/cureus.93234

**Published:** 2025-09-25

**Authors:** Junaid Ayaz Khan, Waniza Ali, Shalika Gunasekaran, Muhammad Haris, Syeda Rafia Zehra, Ayesha Mansoor, Zubair Ahmed, Amina Choudary, Ahmad Hasan, Muhammad Imran Sharif, Haseeb Haider

**Affiliations:** 1 Internal Medicine, Sun Yat-sen Medical College, Guangzhou, CHN; 2 Internal Medicine, Dow International Medical College, Karachi, PAK; 3 Medicine, Government Medical College, Omandurar Government Estate, Chennai, IND; 4 Internal Medicine, Bakhtawar Amin Memorial Trust Hospital, Multan, PAK; 5 Day Care Medicine, Shifa International Hospital Islamabad, Islamabad, PAK; 6 Special Pathology, Community Medicine, ENT, Eye, Allied Hospital Faisalabad, Faisalabad Medical University, Faisalabad, PAK; 7 Internal Medicine, Richmond University Medical Center, New York, USA; 8 Internal Medicine, Lahore Medical and Dental College, Lahore, PAK; 9 Internal Medicine, Assiut University Faculty of Medicine, Assiut, EGY; 10 General Medicine, Almazroui Medical Center, Abu Dhabi, ARE; 11 Internal Medicine, Islam Medical College, Sialkot, PAK

**Keywords:** cardiovascular risk, interheart risk score, non-smokers, smoking, smoking cessation

## Abstract

Background

Cardiovascular disease (CVD) is one of the significant causes of death worldwide, and smoking is a major causative factor of CVD pathogenesis. Smoking rates are high in Pakistan, and the related cardiovascular risks are concerning. The purpose of this study is to compare cardiovascular risk between smokers and non-smokers in a Pakistani population, addressing an under-researched context in cardiovascular health.

Methods

A cross-sectional study was conducted at hospitals in Sialkot from March to July 2025. Participants: A total of 300 participants were included, using convenience sampling. Demographic, smoking status, and cardiovascular health data were obtained using a self-administered questionnaire. The INTERHEART Modifiable Risk Score (IHMRS) was employed to report cardiovascular risk. Data were analyzed using SPSS v.26, employing descriptive statistics, correlation analysis, and multiple regression models.

Results

Of the sample, 190 (63%) were smokers and 110 (37%) were non-smokers. IHMRS scores were significantly higher (29.95 ± 3.10) in smokers than in non-smokers (26.85 ± 2.90) (p<0.001). The cardiovascular risk factors were closely linked to the number of cigarettes smoked per day and to the duration of smoking. The history of cardiovascular disease and family history of CVD were positively correlated with smoking intensity and duration (p<0.001).

Conclusion

Smoking significantly contributes to an increase in cardiovascular risk in Pakistan, and the cardiovascular risks of smokers are higher. The findings highlight the importance of specific population health measures, such as smoking cessation programs, to lessen the burden of cardiovascular diseases in the population.

## Introduction

Cardiovascular disease (CVD) is one of the most common causes of death in the world, and certain countries such as the USA, China, India, Pakistan, Kenya, and Sweden have disproportional mortality rates [1]. Cigarette smoking is a significant cause of cardiovascular diseases, such as coronary heart disease, stroke, and peripheral vascular disease, and the level of risk depends on the quantity and duration of smoking. Although these risks can significantly decrease with smoking cessation, high risks can persist for years, which makes it essential to include focused prevention and education [2,3].

Cigarette smoking has been associated with cardiovascular disease, and the dose-response relationship between smoking and atherosclerosis has been demonstrated. The cigarette smoke is complicated, and it is challenging to detect particular harmful substances in it; however, research has progressed in the field of recognizing the processes of destruction of the cardiovascular system exposed to smoking [4]. One of the major cardiovascular risk factors is tobacco smoking, and the prevalence is significant among men, particularly the middle-aged. Smoking causes coronary heart disease through endothelial dysfunction, raising inflammation, platelet activity, and oxidative stress, whereby female smokers have a 25% higher risk compared to their male counterparts. Early cessation may decrease the risk of excess mortality by as much as 90% [5,6].

Increased intake of fruits and vegetables leads to a low risk of cardiovascular disease among male smokers, and this has a significant effect in preventing the risk of acute coronary syndrome and overall CVD. There was no such association among former or never smokers, which indicates that the protective role is more pronounced in current smokers [7]. Cigarette Smoking causes a significant increase in cardiovascular risk that is reversible on quitting smoking. The advantages of cessation exceed those of specific secondary preventive interventions, and more widespread use of cessation programs is needed to lower the risks of cardiovascular morbidity and mortality [8].

Non-smokers die later and in better cardiovascular health and have a longer history of being without cardiovascular disease, but they also live longer with cardiovascular disease than smokers. Smokers are more exposed to early-life risks of cardiovascular disease, but non-smokers are more exposed to life risks of coronary heart disease and other heart-related illnesses [9]. Cardiovascular biomarker differences between smokers and non-smokers were significant, as smokers experienced lower HDL cholesterol, higher levels of inflammation, oxidative stress, and platelet activation. These biomarkers may provide an aid in determining the cardiovascular risks associated with tobacco consumption [10].

In Pakistan, nationally representative data indicate that approximately 15% of adults smoke, with prevalence reaching over one-quarter of men, while use among women is minimal. Cardiovascular disease remains the leading cause of mortality, accounting for nearly one-quarter of all deaths in 2019 [11,12]. Situating the present study within this context highlights the urgent need to assess the relationship between smoking and cardiovascular risk in the Pakistani population.

Rationale

Although the relationship between smoking and cardiovascular disease (CVD) has been deeply researched in the global context, the concern of the relative prevalence of cardiovascular risk factors in the Pakistani population between smokers and non-smokers is incredibly neglected, as there is a lack of information on such investigations. Due to the increasing burden of noncommunicable diseases, the prevalence of smoking is alarmingly increasing among younger adults in Pakistan. The literature has demonstrated the adverse impact of tobacco on cardiovascular health; however, little is reflected on as to how smoking relates to non-smoking when several cardiovascular risk factors exist, like hypertension, diabetes, and dyslipidemia, in the Pakistani population. Considering the socioeconomic and cultural backgrounds peculiar to Pakistan (poorly developed healthcare awareness in the population and disparities in access to healthcare), the local cardiovascular risk profile of smokers and non-smokers should also be understood to provide suitable prevention and intervention strategies.

Research is needed to bridge the gap in the literature and provide empirical evidence related to Pakistan and its health setting. Available studies do not typically consider regional differences in lifestyles, nutrition, and access to medical facilities, which significantly influence the prevalence and management of cardiovascular risk factors. Since smoking is a common practice in Pakistan, specifically with younger individuals, it is also vital to realise the extent to which it causes the risk factors of cardiovascular diseases as a public health effort. Such differences can be studied, and this work will contribute not only to the formulation of local health policies but also to educating communities about the cardiovascular risks of smoking and helping healthcare providers design more effective prevention programs that can better resonate with the specific needs of the Pakistani population.

Primary objectives

To compare the distribution of cardiovascular risk factors among smokers and non-smokers in a Pakistani study population.

Secondary objectives

To examine the impact of smoking duration and intensity on cardiovascular risk. To assess the role of demographic and lifestyle factors, including age, gender, and socioeconomic status, in shaping differences in cardiovascular risk between smokers and non-smokers.

## Materials and methods

A cross-sectional study design was used to determine the prevalence and burden of cardiovascular risk factors among smokers and non-smokers. The research was conducted at several healthcare providers in Pakistan, including public and private hospitals in Sialkot. These hospitals were selected because they represent a diverse range of socioeconomic and cultural backgrounds, offering a more comprehensive insight into the cardiovascular risk profile of the population.

Structured self-administered questionnaires were used to collect data on various cardiovascular risk factors, which included hypertension, hyperlipidemia, diabetes, and lifestyle-related attributes such as smoking, including the intensity and duration of smoking. Patients were approached during routine clinical visits to the general medicine and cardiology departments. All research participants were informed about the study's aim and methodology by trained research assistants, and therefore, were able to understand the research. The participants were informed and signed the consent forms either in writing or verbally, according to their wishes and literacy level. The participants completed the questionnaires themselves; however, for illiterate or semi-literate respondents, members of the research team assisted in the training. This methodology ensures the collection of trustworthy information while respecting cultural sensitivities and maintaining participant comfort and well-being.

Sampling strategy and population size

This study used a sample size calculated using a World Health Organization (WHO) formula, according to which the proportion of a population is computed based on the confidence interval (95%) and margin of error (5%). The minimum sample size was calculated to be 384 respondents, with an assumed population proportion of 0.5, to maximize variation in the results. Considering all these parameters, it was clear that 385 individuals would be targeted, and allowances were considered accordingly to accommodate individuals who did not reply or had incomplete records [13].To achieve this target, consent was obtained during regular outpatient visits to cardiology and general medicine clinics at Sialkot hospitals (both government and privately-owned). Convenience non-random sampling was used, allowing for the inclusion of all patients visiting the clinics during the study period as participants. Convenience sampling was chosen due to its feasibility and accessibility, allowing for timely data collection from clinical settings during the study period. However, we acknowledge that this method may introduce selection bias and limit the generalizability of the findings beyond the study population. The response rate

was approximately 88% among the 385 people approached to participate in the survey, with 340 accepting participation. However, 40 responses were not analyzed due to incomplete information or ineligibility of the reactions to the study. This resulted in a final sample size of 300 individuals, a number deemed sufficient to make meaningful statistical comparisons between smokers and non-smokers.

Table 1 shows the inclusion and exclusion criteria applied by the research to select participants.

**Table 1:** Inclusion and Exclusion Criteria

Inclusion Criteria	Exclusion Criteria
Adults between 18 and 65 years who undergo regular outpatient care at a group of hospitals.	Participants who have missing data or decline to proceed.
Smokers and non-smokers who volunteered to be participants.	Those whose mental or cognitive condition makes them unable to complete the questionnaire.
Capacity to comprehend and give a written or verbal informed consent.	Pregnant women or any individuals with conditions that could interfere with the goals of the study.
No current diagnosis of a severe or advanced chronic disease (e.g., renal failure, cancer).	People who are already receiving treatment for acute cardiovascular incidents (e.g., heart attack or stroke).

Data collection tools

A structured questionnaire was developed, consisting of two main sections: demographic data and cardiovascular risk factors, to collect comprehensive and necessary data for this study. The main aim of the questionnaire was to estimate the prevalence of cardiovascular risk factors among smokers and non-smokers and identify any relationships between risk factors and being a smoker. All standardized instruments were adopted in their original English language to achieve consistency and reliability. The questionnaire, offered in English, did not necessitate cultural or linguistic customization.

Demographic information

The first section of the questionnaire was designed to collect demographic and clinical data, including information related to smoking. The purpose of this section was to obtain information needed to conduct subgroup analysis and establish the relationship between the demographic factors, smoking status, and the existence of cardiovascular risk factors. The categories of variables obtained were age, gender, marital status, education level, occupation, smoking status (smoker or non-smoker), daily cigarette intake, and smoking duration. Further, the presence of a history of cardiovascular diseases, diabetes, and hypertension in the family was reported. These variables were incorporated to provide a comprehensive view of plausible risk factors that may contribute to the incidence of cardiovascular disease, as well as the extent of these risk factors in smokers and non-smokers. Additional demographic details of the study participants are provided in Appendix 1.

INTERHEART Modifiable Risk Score (IHMRS)

The second section of the questionnaire used the IHMRS to ascertain the cardiovascular risk factors of the participants. Developed in 2010 and based on the original INTERHEART study published by Yusuf and colleagues, IHMRS is an estimator of the probability of experiencing cardiovascular difficulties related to the level of modifiable lifestyle risk factors. These include smoking, hypertension, diabetes, weight and diet, physical activity, stress, and alcohol. The IHMRS has been established as a credible determinant of cardiovascular risk among various populations, including South Asians, and its efficacy has been demonstrated to have an area under the receiver operating characteristic curve (AUC) of greater than 0.75. It indicates that this tool has high potential to differentiate between high- and low-risk groups in the development of cardiovascular disorders. IHMRS helps measure cardiovascular risk because it is easy to apply, very reliable, and can be easily used in both clinical and research settings [14]. Permission to use the questionnaire was obtained via email from the authors. The detailed items of the IHMRS are provided in Appendix 2.

Procedure

The current study was conducted in the outpatient departments of government and non-government hospitals in Sialkot from March to July 2025. The participants were selected through regular visits to the clinics. After their consent, they were given a questionnaire to consider the cardiovascular risk factors and smoking.

Participants were either allowed to complete the questionnaire independently or with the assistance of a trained research assistant, after obtaining informed consent. The research assistants read questions verbatim without interpretation or prompting, and participants’ answers were recorded as stated, to minimize interviewer bias. By using this strategy, the varying degrees of literacy and preferences of the participants who were to complete the survey were taken into account. There was confidentiality, and responses and answers were made anonymously. The study was designed in a manner that upheld ethical practices and considered the cultural sensitivities and diverse needs of the participants' population.

Analytical approach

IBM SPSS Statistics version 26 (IBM Corp., Armonk, New York, USA) was used to perform the data analysis. The demographic characteristics of the participants were described and summarized using descriptive statistics, which included frequencies, percentages, means, and standard deviations. The Kolmogorov-Smirnov and Shapiro-Wilk tests were used to evaluate whether the data were normally distributed. A correlation analysis using the Pearson method was conducted to study the relationship between smoking behaviors, cardiovascular disease variables, and the IHMRS. The IHMRS of smokers and non-smokers was compared using independent samples t-tests.

In contrast, one-way Analysis of Variance (ANOVA) was performed to analyze the variation in risk scores among groups with different smoking duration periods and age groups. We used multiple linear regression analysis to determine the predictors of the IHMRS, smoking status, number of cigarettes smoked, duration of smoking, Diagnosis of cardiovascular disease, and family history of cardiovascular disease. Also, a Chi-square examination was done to understand the relationship between the number of cigarettes taken daily and family history of cardiovascular disease. All tests were conducted at a p<0.05 significance level to identify the most important variables affecting cardiovascular risk, as determined by the IHMRS.

Ethical protocols

The research was conducted in accordance with the ethical guidelines for research involving human subjects. The research protocol was deemed ethically approved by the Institutional Review Board (IRB) of Islam Medical College IMDC/IRB/25/4365. Before data collection, all participants were thoroughly informed of the study's goals, procedures, and potential risks. Informed consent was obtained, and participants could withdraw from the study at any time without repercussions. Confidentiality was ensured by anonymising all participant data when entering, handling, and analysing it. Participants with incomplete responses on key variables were excluded from relevant analyses. After applying these exclusions, the final sample size was 300, which is reported in the Results. No data imputation was performed. The study adhered to the principles of respect for the dignity and privacy of participants, as well as other ethical guidelines.

## Results

Table 2 presents the demographic profile of the subjects (N=300). Most participants were men (N=238, 79%), and 62 (21%) were female. The age distribution revealed that 110 (37%) of the participants fell within the 18 to 25-year age bracket, and 80 (27%) were aged between 46 and 55 years. In terms of marital status, the majority of the respondents were married (N=143, 48%), 69 (23%) were single, and 70 (23%) were divorced. A considerable number of respondents were primary school graduates (N=113, 38%), and a substantial number were employed (N=109, 36%). A majority (N=190, 63%) were smokers, and most of the smokers (N=148, 49%) smoked between five and 10 cigarettes a day. The physical activity levels differed, with 114 (38%) indicating that they rarely engaged in physical exercise. As far as medical conditions are concerned, diabetes and high cholesterol were the most common (N=91, 30%, and N=92, 31%, respectively). A history of cardiovascular disease was reported by 186 (62%) individuals, and 182 (61%) had a family history of similar diseases.

**Table 2:** Demographic Characteristics of Participants (N=300) f: frequency; %: percentage; Values are presented as N (%), N=300; No statistical comparisons were performed for demographic variables in this table.

Variable	f (N)	%
Age	-	-
18–25 years	110	37
26–35 years	45	15
36–45 years	30	10
46–55 years	80	27
56–60 years	20	7
60 years & above	15	5
Gender	-	-
Male	238	79
Female	62	21
Marital status	-	-
Single	69	23
Married	143	48
Divorced	70	23
Widowed	18	6
Educational level	-	-
No formal education	65	22
Primary school	113	38
secondary school	76	25
Undergraduate	38	13
Postgraduate	8	3
Occupation	-	-
Student	49	16
Employed	109	36
Self-employed	88	29
Unemployed	40	13
Retired	14	5
Smokers vs Non-smokers	-	-
Non-smokers	110	37
Smokers	190	63
If you smoke, how many cigarettes do you smoke per day?	-	-
Less than 5	80	27
5-10	148	49
11-20	46	15
21-30	18	6
More than 30	8	3
For how many years have you been smoking?	-	-
Less than 1 year	123	41
1-5 years	109	36
6-10 years	44	15
11-20 years	19	6
More than 20 years	5	2
How often do you engage in physical activity (e.g., walking, running, exercising)?	-	-
Never	107	36
Rarely (1–2 days per week)	114	38
Sometimes (3–4 days per week)	61	20
Often (5 or more days per week)	18	6
Do you have any of the following medical conditions?	-	-
Hypertension (blood pressure)	61	20
Diabetes (Type 1 or 2)	91	30
High cholesterol	92	31
Heart disease	36	12
Stroke	16	5
None	4	1
Have you ever been diagnosed with any cardiovascular diseases (e.g., heart disease, stroke)?	-	-
No	114	38
Yes	186	62
Do you have a family history of cardiovascular disease?	-	-
No	118	39
Yes	182	61

Table 3 presents the results of the normality test for the IHMRS questionnaire. The Kolmogorov-Smirnov and Shapiro-Wilk tests yielded p-values greater than 0.05, indicating that the IHMRS scores follow a normal distribution. The p-values are greater than 0.05; therefore, this shows that the data are typically distributed.

**Table 3:** Tests of Normality for IHMRS Questionnaire IHMRS: INTERHEART Modifiable Risk Score; df: degree of freedom; Values are presented as test statistic (df=300), with corresponding p-values; A p-value >0.05 was considered statistically significant, indicating normal distribution; N=300.

Variables	Kolmogorov-Smirnov			Shapiro-Wilk		
	Statistic	df	p	Statistic	df	p
IHMRS Questionnaire	0.045	300	0.200	0.992	300	0.136

Table 4 shows Pearson correlation coefficients between smoking causes and history of cardiovascular disease and IHMRS as correlates of smoking among smokers. The number of cigarettes smoked per day had significant correlations with other variables, such as the duration of smoking, a diagnosis of cardiovascular disease, family history of cardiovascular disease, and the IHMRS (all p<0.001). Similarly, years of smoking had highly significant positive correlations with a history of cardiovascular disease, family history, and IHMRS. Smoking behavior was the most strongly correlated with a diagnosis of cardiovascular disease and the IHMRS, indicating that cardiovascular health outcomes depend heavily on smoking behavior.

**Table 4:** Pearson Correlations Between Smoking Intensity, Cardiovascular History, and IHMRS Among Smokers IHMRS: INTERHEART Modifiable Risk Score; Values represent Pearson correlation coefficients (r) between continuous variables; p<0.01 (2-tailed) was considered statistically significant and is denoted with double asterisks (**).

Variable	1	2	3	4	5	p
If you smoke, how many cigarettes do you smoke per day?	-	0.598^**^	0.472^**^	0.401^**^	0.522^**^	<0.001^**^
For how many years have you been smoking?	-	-	0.433^**^	0.378^**^	0.457^**^	<0.001^**^
Have you ever been diagnosed with any cardiovascular diseases (e.g., heart disease, stroke)?	-	-	-	0.512^**^	0.481^**^	<0.001^**^
Do you have a family history of cardiovascular disease?	-	-	-	-	0.445^**^	<0.001^**^
IHMRS	-	-	-	-	-	<0.001^**^

Table 5 presents the Pearson correlation coefficients among physical activity, medical conditions, family history of cardiovascular disease, and the IHMRS. The frequency of physical activity was significantly and positively correlated with all other factors, including the occurrence of medical conditions, family history of cardiovascular disease, and the IHMRS (all p<0.001). The correlation between the presence of medical conditions and family history of cardiovascular disease (r=0.33) shows the most substantial impact, as it indicates that people with a family history of cardiovascular disease are more likely to have existing medical conditions, increasing their risk. In addition, the IHMRS was positively related to medical conditions and family history of cardiovascular disease, which revealed the contribution of these factors towards cardiovascular risk.

**Table 5:** Pearson Correlations Between Physical Activity, Medical Conditions, Family History of Cardiovascular Diseases, and IHMRS (N=300) IHMRS: INTERHEART Modifiable Risk Score; Values represent Pearson correlation coefficients (r) between continuous variables; p<0.01 (2-tailed) was considered statistically significant and is denoted with double asterisks (**).

Variable	1	2	3	4	p
How often do you engage in physical activity (e.g., walking, running, exercising)?	-	0.28^**^	0.24^**^	0.31^**^	<0.001^**^
Do you have any of the following medical conditions?	-	-	0.33^**^	0.27^**^	<0.001^**^
Do you have a family history of cardiovascular disease?	-	-	-	0.29^**^	<0.001^**^
IHMRS	-	-	-	-	<0.001^**^

Table 6 shows a comparison of the IHMRS questionnaire between smokers and non-smokers. The mean score of the IHMRS was found to be significantly higher in smokers, with a mean IHMRS score of 29.95 (SD=3.10), compared to non-smokers, who had a mean IHMRS score of 26.85 (SD=2.90). An independent samples t-test revealed a significant difference (t=8.871, p<0.001) with a large effect size (Cohen's d=1.02). Consequently, the results indicated that smoking was associated with an increased cardiovascular risk, as measured by the IHMRS. The 95% confidence interval limits on the mean difference were 2.41 and 3.79, indicating a significant difference in the mean risk scores between the two groups.

**Table 6:** Comparison of IHMRS Questionnaire Between Smokers and Non-smokers (N=300) IHMRS: INTERHEART Modifiable Risk Score; Values are presented as mean±standard deviation; Independent samples t-tests were conducted to compare participants with and without smoking; Group sizes are shown as N (%); Reported statistics include p-values, t-values, 95% Confidence Intervals (CI), and effect sizes (Cohen’s d); A p-value <0.01** was considered statistically significant, N=300.

Variable	Smoker (N=190;63%) M±S.D	Non-smoker (N=110;37%) M±S.D	t	p	Cl 95% LL	UL	Cohen's D
IHMRS Questionnaire	29.95±3.10	26.85±2.90	8.871	<0.001^**^	2.41	3.79	1.02

Table 7 shows means, standard deviations, and a one-way ANOVA of the IHMRS questionnaire by length of smoking. IHMRS scores mean was higher with prolonged smoking duration, and the lowest score was recorded among participants who had smoked for less than one year (M=25.80, SD=2.90), whereas the highest score was realized in those who had smoked for more than 20 years (M=33.40, SD=1.82). According to ANOVA data, the effect of smoking duration had a significant impact on IHMRS scores (F=26.88, p<0.001), with a moderate effect size (η²=0.267). This implies that the more people smoke, the more they are at risk of cardiovascular damage, calculated in terms of the IHMRS, which appears to be a cumulative consequence of smoking.

**Table 7:** Means, Standard Deviations, and ANOVA Results for IHMRS by Smoking Duration (N=300) IHMRS: INTERHEART Modifiable Risk Score; Data are presented as mean±standard Deviation (M±SD); Group sizes are shown as N (%); One-way ANOVA was conducted to examine the effect of smoking duration on IHMRS; A p-value <0.01** was considered statistically significant; η² represents partial eta-squared effect size.

Smoking Duration	Less than 1 year (N=123;41%) M±S.D	1-5 years (N=109;36%) M±S.D	6-10 years (N=44;15%) M±S.D	11-20 years (N=19;6%) M±S.D	More than 20 years (N=5;2%) M±S.D	p	F (4,295)	η2
IHMRS Questionnaire	25.80±2.90	27.40±2.70	29.10±3.00	31.20±2.80	33.40±1.82	<0.001^**^	26.88	0.267

Table 8 presents the means, standard deviations, and outcomes of a one-way ANOVA comparing smoking among smokers in three age categories: 18-25, 26-35, and 46-55 years. There were significant differences detected in the number of cigarettes smoked a day (p=0.003) and the years the subject has smoked (p=0.014). Notably, among the smokers aged 46-55 years, they indicated that they smoked an average of 3.00 cigarettes in a day as opposed to their younger counterparts. Additionally, the years of smoke were more prevalent in the 46-55 age category (M=2.05 years). The effect sizes (η²) indicate moderate differences, such as the number of cigarettes smoked per day (η²=0.076), which suggests that age influences smoking intensity and duration in these participants.

**Table 8:** Means, Standard Deviations, and ANOVA Results for Smoking Behavior Among Current Smokers by Age Group Data are presented as mean±standard deviation (M ± SD); Group sizes are shown as N (%); One-way ANOVA was conducted to compare smoking status and IHMRS by age groups; A p-value <0.01 was considered statistically significant; η² represents partial eta-squared effect size.

Age	18–25 years (N=101;53%) M±S.D	26–35 years (N=29;15%) M±S.D	46–55 years (N=60;32%) M±S.D	p	F (2,143)	η2
If you smoke, how many cigarettes do you smoke per day?	2.14±0.930	2.00±0.739	3.00±0.408	0.003^**^	5.88	0.076
For how many years have you been smoking?	1.81±0.906	1.67±0.651	2.05±0.324	0.014^*^	4.38	0.058

Table 9 shows notable differences in physical activity, medical conditions, and the IHMRS among different age groups. Younger people (18-35 years) had a higher rate of physical activity than older groups, and those least involved in physical activity were those in the 56-60 age bracket. Older people, especially those aged 56 years and older, had more medical conditions. The IHMRS for the 56-60 age group was the highest, indicating that older individuals are at a high cardiovascular risk. These findings suggest that the level of physical activity, as well as the presence or absence of medical conditions, is highly dependent on age, which can help mitigate the cardiovascular risk based on the IHMRS.

**Table 9:** Means, Standard Deviations, and ANOVA Results for Physical Activity, Medical Conditions, and IHMRS by Age Group IHMRS: INTERHEART Modifiable Risk Score; Data are presented as mean±standard Deviation (M ± SD); Group sizes are shown as N (%); One-way ANOVA was conducted to compare physical activity, medical conditions, and the IHMRS by age groups; A p-value <0.01 was considered statistically significant; η² represents partial eta-squared effect size.

Variable & Age Group	18–25 years (N=110;37%) M±S.D	26–35 years (N=45;15%) M±S.D	36-45 years (N=30;10%) M±S.D	46-55 years (N=80;27%) M±S.D	56-60 years (N=20;7%) M±S.D	60 years & above (N=60;5%) M±S.D	p	F	η2
How often do you engage in physical activity?	1.95±0.876	2.04±1.048	2.39±0.894	3.00±0.075	1.00±0.050	2.18±0.561	<0.001^**^	12.5	0.145
Do you have any of the following medical conditions?	2.56±1.175	2.54±1.261	2.00±0.050	2.10±0.105	4.00±0.050	3.19±0.414	<0.001^**^	10.25	0.122
IHMRS	27.63±3.124	28.20±2.535	26.59±1.949	26.41±0.105	30.00±0.050	28.09±1.021	<0.001^**^	9.20	0.111

Table 10 shows the outcome of a multiple linear regression analysis to predict total IHMRS based on smoking and cardiovascular disease variables. The intercept value of 25.412 had a significant correlation with the constant term (IHMRS) (p<0.001). Smoking status was also a decisive factor in predicting higher risk scores (B=1.124, β=0.185, p<0.001), as well as the number of cigarettes smoked per day (B=0.482, β=0.148, p=0.001) or the number of years that a person smoked (B=0.395, β=0.126, p=0.002). Having a history of cardiovascular disease (B=1.002, β=0.160, p<0.001) and having a family history of cardiovascular disease (B=0.872, β=0.140, p<0.001) also proved to be significant predictors of increasing IHMRS. The p-values of all predictors were below 0.05, indicating that both cardiovascular disease history and smoking behaviour contribute significantly to defining cardiovascular risk, as quantified by the IHMRS.

**Table 10:** Multiple Regression Predicting Total IHMRS from Smoking and Cardiovascular Disease Variables (N=300) IHMRS: INTERHEART Modifiable Risk Score; Multiple linear regression was conducted to identify predictors of IHMRS. The results included unstandardized coefficients (B), 95% confidence intervals (CI), standard error (SE), standardized beta coefficients (β), and p-values. A p-value <0.05 was considered statistically significant. The study included 300 participants (N=300).

Predictor	B	SE	β	t	p	95% CI LL	UL
Constant IHMRS	25.412	1.005	—	25.29	<0.001^**^	23.44	27.38
Do you smoke?	1.124	0.285	0.185	3.94	<0.001^**^	0.563	1.685
If you smoke, how many cigarettes do you smoke per day?	0.482	0.142	0.148	3.39	0.001^**^	0.203	0.761
For how many years have you been smoking?	0.395	0.128	0.126	3.09	0.002^**^	0.143	0.647
Have you ever been diagnosed with any cardiovascular diseases (e.g., heart disease, stroke)?	1.002	0.261	0.160	3.84	<0.001^**^	0.488	1.516
Do you have a family history of cardiovascular disease?	0.872	0.238	0.140	3.66	<0.001^**^	0.403	1.341

Table 11 shows the relationship between the number of cigarettes smoked a day and a history of cardiovascular illness in the family. Chi-square test identified that the correlations between the number of cigarettes smoked per day and family history of cardiovascular disease were statistically significant (χ²=12.08, p=0.011). The proportion of the participants who smoked less than five cigarettes a day was higher in the group with a family history of cardiovascular disease (N=57, 31%) than in the group without a family history (N=23, 20%). Also, a larger proportion of respondents with no family history of cardiovascular disease were smokers taking 5-10 cigarettes daily (N=54, 47%) than respondents with a family history (N=94, 50%). The significant value of p indicates that there is a connection between daily smoking and the existence of a family history of cardiovascular disease.

**Table 11:** Association Between Daily Cigarette Consumption and Family History of Cardiovascular Disease (N=300) Data are presented as N (%); the Chi-square test was used to assess the relationship between daily cigarette consumption and the frequency of family history of cardiovascular diseases; Statistical significance was considered at p<0.05.

If you smoke, how many cigarettes do you smoke per day?	No	Yes	Total	χ² (df=4)	p
Less than 5	23 (20%)	57 (31%)	80 (27%)	-	-
5-10	54 (47%)	94 (50%)	148 (49%)	-	-
11-20	25 (22%)	21 (11%)	46 (15%)	-	-
21-30	6 (5%)	12 (6%)	18 (6%)	-	-
more than 30	6 (5%)	2 (1%)	8 (3%)	-	-
Total	114	186	300	12.08	0.011^*^

## Discussion

In the current study, the researcher sought to analyse the cardiovascular risk factors among smokers and non-smokers in Pakistan. The findings reveal key disparities in cardiovascular health between these two groups, as measured by the IHMRS. Our study showed a strong correlation between the number of cigarettes smoked per day and smoking duration, where a longer duration of smoking was associated with a history of cardiovascular disease. Similarly, a study has noted the significance of proper assessment of smoking heaviness, whereby the findings indicate that the duration and quantity of smoking are critical in the view of cardiovascular risk [15]. We observed in our study that the heavier the smoking base, the more the family history of cardiovascular disease, implying that there was an interaction between smoking and genetic factors. It concurs with an earlier study that reported a robust family history of coronary disease combined with cigarette smoking significantly increased the risk of getting coronary artery disease, with chances of the disease being highly increased in the presence of both [16]. In our research, there is a correlation between smoking more cigarettes per day and the history of cardiovascular disease, together with a high IHMRS, determining higher cardiovascular risk. On the same vein, a study conducted revealed a positive relationship between smoking and increased levels of inflammatory markers and homocysteine, further supporting the idea that smoking increases cardiovascular risk, even regarding more unconventional risk factors [17]. Moreover, we also discovered that long duration of smoking was linked with a prior history of cardiovascular disease, and this was in line with the findings that long-term smoking led to a substantial risk of cardiovascular diseases over time [17]. Our analysis shows that there was a strong relationship between smoking behavior and prior cardiovascular disease, as well as a higher IHMRS, indicating that smoking poses high cardiovascular risks. Likewise, the fact that cigarette smoking was a significant determinant of both morbidity and mortality in cardiovascular disease has been soundly established, and smoking was found to elevate the risk of coronary heart disease, stroke, and peripheral vascular disease [2].

In our study, we found that physical exercise was associated with improved health conditions and a lower prevalence of cardiovascular risk among individuals with medical conditions. Another study conducted among patients with chronic diseases found that greater exercise was associated with improved functioning and well-being, suggesting the utility of physical activity in managing chronic diseases [18]. Additionally, physical activity in our study was associated with improved cardiovascular health outcomes. Similarly, previous studies have established links between low physical activity, high body mass index (BMI), and systolic blood pressure (SBP), as well as the risk of developing hypertension, and that frequent exercise is essential in minimizing cardiovascular risk [19]. We further found out that past medical disorders, including hypertension or diabetes, were related to the IHMRS, which was high, indicating increased cardiovascular risk. Likewise, other literature has also revealed that hypertension in diabetic patients contributes to significant cardiovascular disease risks and thus the importance of its control to limit cardiovascular morbidity [20]. Family history of cardiovascular disease was closely associated with cardiovascular risk in our study, as was reported in a survey that assessed premature coronary heart disease and showed that family history and risk were dose coincidental. These two studies highlight the importance of incorporating family history in determining cardiovascular risks and prevention [21]. We also observed in our research that a family history of cardiovascular disease contributed to increased diabetes as well as CVD risk. In the same way, other studies have revealed that children with a family history of such disorders are already at a higher risk, underlining the necessity of emphasizing the family history in case of initial screening and prevention efforts [22].

Smokers in our study had significantly higher IHMRS (indicating an elevated cardiovascular risk), and this pattern in our study is correlated with the fact that several research studies have shown direct dependence of cardiovascular disease and smoking. The dose-response correlation of the degree of smoking and cardiovascular risk makes further arguments on the idea that smoking is one of the significant factors that lead to atherosclerosis and other cardiovascular diseases [4].

Our results demonstrated that the longer the smoking, the higher the cardiovascular risk, which was evident through IHMRS. It is consistent with what has already been written on this, the prolonged effect of smoking, including dysfunction of the endothelium and a higher predisposition to cardiovascular disease, focusing on the accrued harm of smoking with time [6].

Our results are consistent with the available literature, which indicates that age is one major factor that affects smoking behavior. In our research, the number of smoked cigarettes per day was higher among the smokers of 46-55 years old compared with the smokers of the younger age groups, in agreement with earlier research, which indicates that younger individuals smoke a lower number of cigarettes per day. Those who initiate smoking earlier in life are more likely to develop heavy smoking patterns over time [23]. Our study established that smokers of older age groups were likely to have a high smoking experience, which supports other studies showing that the sooner a person initiates smoking, the more difficult it becomes to quit. These findings indicate that the age of smoking initiation might be an essential variable in the conceptualization of how people smoke and in the development of effective cessation programs [24].

We observed that younger individuals were more physically active, while older individuals were less physically active in our study. Likewise, it has been demonstrated that physical activity typically decreases at older ages, which places individuals at a greater risk of developing conditions such as obesity, diabetes, and high blood pressure, which further poses a cardiovascular risk [25]. Our study showcased a higher prevalence of various historical medical conditions, such as hypertension and diabetes, amongst the older respondents, with the highest probability of having multiple medical issues found at the age range of 56-60 years. The same trend was observed in the earlier study, in which the prevalence of diseases or conditions like diabetes, hypertension, and obesity was on the rise with age [26]. Furthermore, in our study, older individuals with a higher IHMRS were found to have a higher cardiovascular risk. This is consistent with other studies indicating that the prevalence of CVD and associated risk factors increases with age. Therefore, there is a need to ensure special CVD prevention and management strategies among older people [27].

Smoking status emerged as a strong predictor of increased cardiovascular risk in our study, consistent with other literature stating that smoking increases the risk of atherosclerosis and cardiovascular disease [4]. We discovered in our research that the number of cigarettes smoked daily was a leading indicator of CV risk because greater cigarette consumption in a day was associated with a higher IHMRS. Likewise, the prospective study revealed that even smoking one to four cigarettes per day substantially increased the probability of the development of ischaemic heart disease and other health complications, which supported the necessity of viewing even light smoking as a critical risk factor [28]. In our study, we noted that years of smoking have provided a significant contribution to the IHMRS in individuals reflecting high cardiovascular risk. Likewise, an analysis on smoking cessation also identified that the beginning age at which people started smoking and the number of years spent smoking were significant predictors of smoking persistence, which once again emphasizes the chronic effects of smoking on health outcomes [24]. A history of cardiovascular disease, as well as a family history of cardiovascular disease, in our study were both strong predictors of higher IHMRS, which represent an increased cardiovascular risk. This concurs with an earlier study, whose findings also revealed a high relationship between family history and perceived risk; however, changes in health-related behavior were not as consistently present in response to this awareness [29].

In our study, we discovered that when people had a history of cardiovascular disease in their families, they smoked fewer than five cigarettes daily. This follows the results of earlier research, which also reported a dose-dependent relationship between the intensity of smoking and inflammatory markers, revealing that smoking cessation results in an improvement of cardiovascular health with time [30].

While the association between smoking and cardiovascular risk is well established globally, this study contributes by examining the relationship in a Pakistani population, which remains under-researched. By applying the IHMRS in this setting, the study provides context-specific insights that are essential for guiding locally relevant public health interventions.

Limitations

Several limitations must be noted in this research. First, the causal relationship between smoking and cardiovascular risk factors cannot be determined because of the cross-sectional design. The causal effects of tobacco on cardiovascular health would be more accurately determined using longitudinal studies. Second, the research sample was based on self-reports on smoking status and cardiovascular risk factors, which can provide recall bias or inaccuracies. Similarly, physical activity was assessed through self-reported categories, which may be subject to recall bias and social desirability bias, as participants might have over- or under-reported their actual activity levels. Another limitation is that the IHMRS includes smoking as a variable, which may inflate associations when smoking is also analyzed as a predictor. Moreover, other potential confounding factors, such as environmental exposures, psychological stress, and diet, were not evaluated in the research study, which may have influenced cardiovascular risk. Additionally, the sample was predominantly male (79%), which not only limits the applicability of the results to females but also restricts meaningful gender comparisons. The convenience sampling minimizes the chance of the responses generalizing the findings to the entire population, as the respondents were not randomly selected and only those accessible were approached. Lastly, data was only recorded in major cities and may not accurately reflect the numbers of the rural population in Pakistan, where access to healthcare and smoking habits may vary.

Future directions

Future research should focus on establishing a longitudinal cohort to understand the impact of smoking on the long-term cardiovascular outcomes of the Pakistani population. Additionally, it would be beneficial to quantify a broader range of cardiovascular risk factors, such as genetic predispositions, stress levels, and dietary habits, as this would provide a more comprehensive understanding of the cardiovascular risk determinants. The research study may also be scaled by being conducted on a larger sample size and with a larger number of female respondents, thereby making it more representative. Future research should also explore the impacts of smoking cessation programs on cardiovascular risk in Pakistani smokers, which might lead to more effective population health interventions. Future research should focus on a more diverse sample that includes a broader range of representation between urban and rural demographic groups, and that is randomly selected, to surpass the limitations of convenience sampling and provide more suitable external validity.

## Conclusions

In conclusion, the study has presented meaningful findings on the distribution of cardiovascular risk factors among Pakistani smokers and non-smokers. These findings indicate that smokers were associated with higher cardiovascular risk scores, particularly among individuals with a history of smoking and those reporting infrequent but intensive smoking. The results underscore the importance of holistic population health efforts aimed at smoking cessation, which may help in reducing the burden of cardiovascular diseases in the country. Policymakers should consider more comprehensive smoking cessation initiatives, particularly among young adults, as a strategy that could contribute to lowering the long-term cardiovascular risks associated with smoking.

## Appendices

Appendix 1 

**Table 12:** Demographic Information Questionaaire

Items	Responses
Age	-
Gender	-
Marital Status	-
Educational Level	-
Occupation	-
Do you smoke?	-
If you smoke, how many cigarettes do you smoke per day?	-
For how many years have you been smoking?	-
How often do you engage in physical activity (e.g., walking, running, exercising)?	-
Do you have any of the following medical conditions? (Select all that apply)	-
Have you ever been diagnosed with any cardiovascular diseases (e.g., heart disease, stroke)?	-
Do you have a family history of cardiovascular disease?	-

Appendix 2 

**Table 13:** IHMRS Questionnaire IHMRS: INTERHEART Modifiable Risk Score; Data adapted from the IHMRS Questionnaire, developed in 2010 by Yusuf and colleagues [13]. Permission to use the questionnaire was obtained via email from the authors.

Item Domain	Example Response Options
Participant information	Date of birth, date of assessment, sex
Tobacco use	Current smoking status; average number of cigarettes/day; total years of smoking
Passive smoke exposure	Frequency of exposure to second-hand smoke during the past year
Medical history	Presence of diabetes; history of high blood pressure
Psychosocial factors	Frequency of stress at work/home (past year); any episode of persistent low mood ≥2 weeks (past year)
Family history	Biological parents with a prior heart attack
Dietary habits	Daily intake of salty foods/snacks; frequency of deep-fried/fast foods (≥3 times/week); daily fruit intake; daily vegetable intake; meat/poultry consumption (≥2 times/day)
Physical activity	Usual level of activity during leisure time
